# Feasibility of a Traceback Approach for Using Pathology Specimens to Facilitate Genetic Testing in the Genetic Risk Analysis in Ovarian Cancer (GRACE) Study Protocol

**DOI:** 10.3390/jpm11111194

**Published:** 2021-11-13

**Authors:** Tia L. Kauffman, Yolanda K. Prado, Ana A. Reyes, Jamilyn M. Zepp, Jennifer Sawyer, Larissa Lee White, Jessica Martucci, Suzanne Bianca Salas, Sarah Vertrees, Alan F. Rope, Sheila Weinmann, Nora B. Henrikson, Sandra Soo-Jin Lee, Heather Spencer Feigelson, Jessica Ezzell Hunter

**Affiliations:** 1 Department of Translational and Applied Genomics, Kaiser Permanente Northwest, Center for Health Research, 3800 N. Interstate Ave., Portland, OR 97227, USA; tia.l.kauffman@kpchr.org (T.L.K.); yolanda.k.prado@kpchr.org (Y.K.P.); ana.a.reyes@kpchr.org (A.A.R.); jamilyn.m.zepp@kpchr.org (J.M.Z.); sarah.vertrees@kpchr.org (S.V.); alan@genomemedical.com (A.F.R.); sheila.weinmann@kpchr.org (S.W.); 2 Institute for Health Research, Kaiser Permanente Colorado, 2550 S. Parker Road, Suite 200, Aurora, CO 80014, USA; jennifer.k.sawyer@kp.org (J.S.); Larissa.L.White@kp.org (L.L.W.); heather.s.feigelson@kp.org (H.S.F.); 3 History & Sociology of Science Department, University of Pennsylvania, Cohen Hall Suite 303, 249 South 36th Street, Philadelphia, PA 19104, USA; jmartucc@upenn.edu; 4 Research Data and Analysis Center, Kaiser Permanente Northwest, Center for Health Research, 3800 N Interstate Ave., Portland, OR 97227, USA; suzanne.b.salas@kpchr.org; 5 Genome Medical, 701 Gateway Boulevard, Ste. 380, South San Francisco, CA 94080, USA; 6 Kaiser Permanente Washington Health Research Institute, 1730 Minor Ave., #1600, Seattle, WA 98101, USA; nora.b.henrikson@kp.org; 7 Department of Medical Humanities and Ethics, Division of Ethics, Columbia University, 630 West 168th Street, PH 1525, New York, NY 10032, USA; sandra.lee@columbia.edu; 8 Genomics, Ethics and Translational Research Program, RTI International, 3040 East Cornwallis Road, Research Triangle Park, NC 27709, USA

**Keywords:** ovarian cancer, traceback testing, cascade testing, post-mortem genetic testing, pathology, hereditary breast, ovarian cancer

## Abstract

Guidelines currently state that genetic testing is clinically indicated for all individuals diagnosed with ovarian cancer. Individuals with a prior diagnosis of ovarian cancer who have not received genetic testing represent missed opportunities to identify individuals with inherited high-risk cancer variants. For deceased individuals, post-mortem genetic testing of pathology specimens allows surviving family members to receive important genetic risk information. The Genetic Risk Assessment in Ovarian Cancer (GRACE) study aims to address this significant healthcare gap using a “traceback testing” approach to identify individuals with a prior diagnosis of ovarian cancer and offer genetic risk information to them and their family members. This study will assess the potential ethical and privacy concerns related to an ovarian cancer traceback testing approach in the context of patients who are deceased, followed by implementation and evaluation of the feasibility of an ovarian cancer traceback testing approach using tumor registries and archived pathology tissue. Descriptive and statistical analyses will assess health system and patient characteristics associated with the availability of pathology tissue and compare the ability to contact and uptake of genetic testing between patients who are living and deceased. The results of this study will inform the implementation of future traceback programs.

## 1. Introduction

Individuals at increased risk of breast and ovarian cancer associated with pathogenic variants in *BRCA1/2* account for up to 10% of breast cancer cases, 15% of ovarian cancer cases, and up to 20% of cases of high-grade serous ovarian cancer, the most aggressive subtype [1,2,3,4]. The prevalence of pathogenic variants in *BRCA1/2* in the general population has been estimated as 0.2–0.3% [5], though recent evidence indicates this prevalence may be as high as 0.5% [6], or about 825,000 individuals in the United States. However, it is estimated that only about 6% of individuals with pathogenic variants in *BRCA1/2* have been identified in the general population [7,8,9,10,11,12]. This results in a significant healthcare gap given that early identification of individuals at increased risk for breast and ovarian cancer allows for implementation of interventions shown to reduce cancer-related morbidity and mortality [5,13,14,15]. Risk-reducing surgery among individuals with *BRCA1/2* pathogenic variants is associated with reductions in breast and ovarian cancer of 85–100% and 69–100%, respectively [13]. Individuals who do not undergo risk-reducing surgery are recommended to have an earlier start on breast cancer surveillance [15], which is associated with detection of breast cancer at an earlier, more treatable stage [16,17]. In addition to saving lives, early identification and intervention is cost-effective [18,19,20]. The Centers for Disease Control and Prevention (CDC) has designated *BRCA1/2* screening as having Tier 1 evidence [21] for reducing cancer morbidity and mortality [22], and the significance of these efforts is highlighted by the Healthy People 2030 objective to “Increase the proportion of females with a family history of cancer who receive genetic counseling for hereditary breast and/or ovarian cancer based on the most recent guidelines [23]”.

In 2007, the National Comprehensive Cancer Network (NCCN) recommended genetic counseling and consideration of genetic testing in *BRCA1/2* for all individuals diagnosed with epithelial non-mucinous ovarian cancer, which includes fallopian tube and primary peritoneal cancers [15]. Review of studies from the decade following that recommendation shows rates of genetic testing in this population are only between 15% and 31% [24,25]. Since the 2007 recommendation, newer research also shows that individuals with ovarian cancer have pathogenic variants in genes other than *BRCA1/2*, and NCCN updated its guideline in 2016 to recommend a gene panel [26].

The rapid progression of ovarian cancer in many patients means that obtaining samples for testing is challenging [27]. Genetic testing can be performed using a variety of biological specimens, including blood, saliva, and even pathology tissue. Pathology specimens typically contain non-tumor tissue (e.g., margins) that can be used for germline genetic testing [28,29]. Recent studies have demonstrated high sensitivity and specificity of detecting germline variants in *BRCA1/2* using formalin-fixed paraffin-embedded (FFPE) specimens from breast and ovarian cancer cases [28,29,30]. Testing existing pathology specimens eliminates the need to collect additional biological specimens and, importantly, makes it possible to provide genetic risk information to families of deceased patients. Testing a specimen from a deceased patient is preferable to testing surviving relatives because a negative result in a surviving relative does not rule out the possibility that the deceased patient harbored a pathogenic variant that may be present among other relatives.

In 2016, the Division of Cancer Prevention and the Division of Cancer Control and Population Sciences of the National Cancer Institute sponsored a workshop of experts to discuss a traceback testing approach to increase the identification of families at increased genetic risk for cancer [24]. This approach specifically addresses a missed opportunity by offering genetic testing to patients with a prior diagnosis of ovarian cancer who did not receive genetic testing, such as when their diagnosis preceded the development of recommendations for genetic counseling in all cases of ovarian cancer. The outcome of this workshop inspired a cooperative agreement funding announcement “To support pilot research projects using a “traceback” approach to genetic testing [individuals] with a personal or family history of ovarian cancer and reaching out to family members to identify unaffected individuals at increased risk for cancer [cascade testing] in different clinical contexts and communities, including racially/ethnically diverse populations [31].” Here we detail the protocol, as of January 2021, of the Genetic Risk Assessment in Ovarian Cancer (GRACE) study, which aims to assess the feasibility of using the tumor registry and pathology specimens for an ovarian cancer traceback approach.

## 2. Materials and Methods

### 2.1. Study Design

GRACE was designed to identify individuals through the tumor registry with a prior diagnosis of ovarian cancer, including fallopian and peritoneal cancers, who could benefit from genetic testing of genes associated with an increased risk of breast and ovarian cancers.

The study will first focus on assessing overarching issues that impact a traceback approach, including ethical and privacy concerns related to identifying individuals with a prior diagnosis of ovarian cancer and offering genetic testing to them and their family members. The ethical and legal implications will be assessed through content and legal analysis of guidance and laws related to protection of health information in the states in which recruitment will take place: Oregon, Washington, and Colorado. Perspectives on perceived risks, benefits, and preferred communication of being identified and offered genetic testing will be assessed using semi-structured interviews among at-risk family members of living and deceased participants. This work will be conducted first and will provide important legal and policy information about how we will be allowed to identify and contact personal representatives of deceased patients. Our planned approach is provided in this manuscript, but our approach may be adjusted based on these learnings. 

This study will implement and evaluate the feasibility of an ovarian cancer traceback testing approach using tumor registries and archived pathology tissue. Once genetic testing results are received, any positive tests results will then have at-risk biological relatives eligible for genetic testing to see if the relative has the same pathogenic variant (cascade testing). Using descriptive and statistical analyses, we will assess health system and patient characteristics associated with availability of pathology tissue and compare the ability to contact living patients or personal representatives of deceased patients, the uptake of genetic testing, and the uptake of cascade testing among at-risk family members between living and deceased participants.

We will establish a project-specific External Advisory Panel (EAP), consisting of patient advocates and experts in relevant fields such as clinical genetics; cancer genetics; oncology; and the ethical, legal, and social implications of genomic medicine. The EAP will be consulted as needed to provide input on study activities such as protocol design, implementation of the study, data analysis and reporting, and dissemination of study findings.

We plan to use normal (non-tumor) tissue captured in pathology samples for testing or allow for a self-collection of saliva for patients who are living. For individuals who are deceased, we will ask their personal representative for permission to test the pathology tissue. Cascade testing will be offered to family members of individuals with a positive test result.

### 2.2. Setting

Eligible participants will be individuals with a female sex listed in the EHR with a diagnosis of ovarian cancer (see Table 1 for specific cancer codes; hereafter referred to as ovarian cancer) between 2008 and 2019 at either of two managed care organizations: KPNW or Kaiser Permanente Colorado (KPCO). 

KPNW and KPCO are integrated health care delivery systems that together provide comprehensive care to over 1.2 million members. The membership of KPNW and KPCO reflects the population in each catchment area, with about 20% and 25% racial/ethnic minority members, respectively, and about 15% with low socioeconomic status (income below poverty level and less than high school education based on census data) in each region. Attrition is low among members, with current annual retention rates of 91% at KPNW and 85% at KPCO. Both regions have tumor registries that track a patient’s entire course of care after a cancer diagnosis, and both regions contain in-house archived pathology specimens dating back at least 10 years. 

### 2.3. Inclusion and Exclusion Criteria

**Figure 1:**
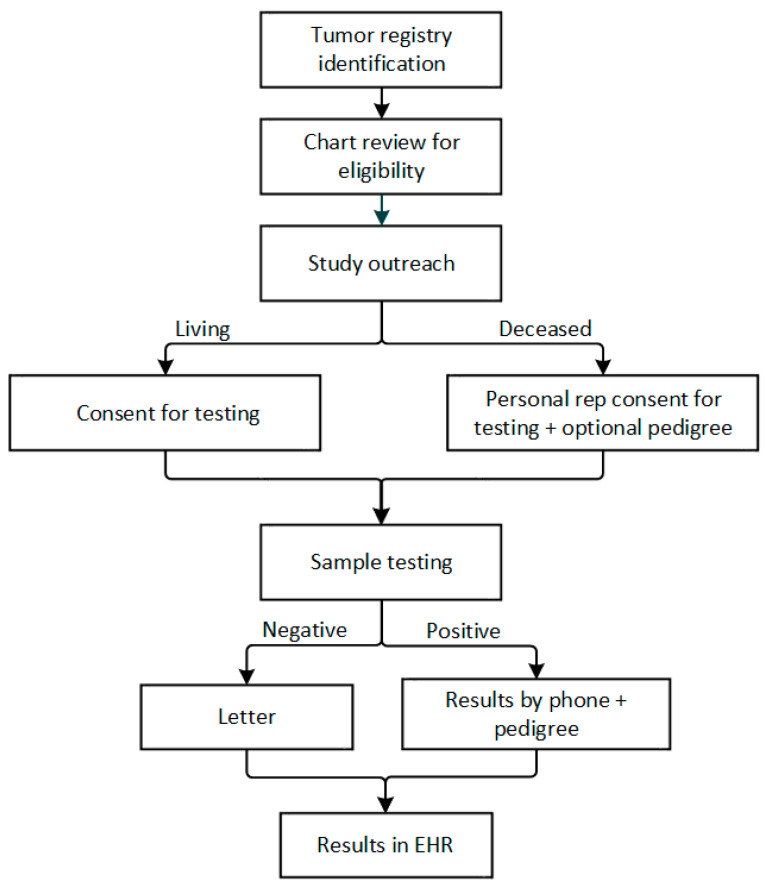
Genetic Risk Assessment in Ovarian Cancer (GRACE) study flow.

GRACE has separate inclusion and exclusion criteria for patients with ovarian cancer enrolling in the study and for family members enrolling to receive cascade testing (Table 1). After identifying adult patients at KPNW and KPCO with a prior diagnosis of ovarian cancer from 2008–2019 and who have not opted out of research activities, we will perform manual chart review on all patients to determine eligibility, including any prior diagnosis of a hereditary cancer syndrome and obtaining any prior genetic testing results, which are typically entered into the electronic health record (EHR) as scanned documents (Figure 1).

We will also query the research-ready database populated by the EHR, where prior genetic testing results may be captured. Patients will be eligible for the study, and therefore genetic testing, if they either have not received genetic testing or if they received genetic testing for *BRCA1/2* only with a negative result. For eligible individuals who are deceased, we will also pre-screen to ensure there is a testable sample available either from (a) pathology tissue from the ovarian cancer surgery, (b) pathology tissue from another surgery if none is available from an ovarian cancer surgery, or (c) a sample in the Northwest biobank (KPNW members only). We will also perform a manual chart review to look for legal documentation of the personal representative and the personal representative’s contact information for all deceased individuals.

### 2.4. Recruitment

#### 2.4.1. Living Patients

Patients with ovarian cancer who are still living based on EHR data will be sent a recruitment letter and fact sheet about the study, by both mail and email (if available), which will include a direct link to a personalized consent document in REDCap [32,33]. A trained research assistant will follow up with a phone call to the patient to confirm eligibility and answer any questions for patients who do not respond to the mailing or email recruitment outreach.

#### 2.4.2. Personal Representatives

Here, we present our current planned approach to contact personal representatives to potentially consent to allow genetic testing of pathology specimens of individuals who are deceased. Our approach may be modified based on our findings from the legal and regulatory review. Our first recruitment outreach to the personal representative will be by letter, to minimize the emotional impact of a subsequent phone call to assess interest. The personal representative will be invited to contact the study to discuss participation, ask further questions, or to decline participation. If the personal representative has not contacted the study within a 2–4-week window, we will contact the personal representative by phone, if possible. When we have contact with the personal representative, per local and federal laws and policies, we will verify they are the personal representative by requesting documentation that they are the personal representative such as a will or a letter that confirms they are the executor. Once verification is obtained, we will explain the study, confirm the eligibility of the deceased proband, and answer any questions. Due to the more sensitive nature of contacting a personal representative of a deceased patient, this recruitment will be conducted by a genetic counselor. Given the proband is deceased, the benefit to testing their pathology tissue is to provide the genetic risk information to at-risk relatives and offer them cascade testing. Thus, the genetic counselor will discuss with the personal representative whether there are any at-risk family members to benefit from cascade testing. This will be mentioned at the time of consent and discussed in more detail at the time of results disclosure (if positive).

#### 2.4.3. Informed Consent

Informed consent will be obtained from all subjects involved in the study. Patients and personal representatives will either be provided a personalized link to an electronic consent in REDCap or mailed a paper copy of the consent form, depending on preference.

### 2.5. Study Procedures

#### 2.5.1. Genetic testing

After obtaining informed consent from living participants or the personal representative of a deceased patient, archived pathology samples with normal tissue from the patient with ovarian cancer will be requested in accordance with requirements of the commercial genetic testing laboratory, detailed in the site local procedure. In some cases, a biospecimen stored in the Northwest Biobank may be used for participants consented by the personal representative (if available) or self-collected saliva sample will be requested from living patients if that is their preferred testing sample or if genetic testing is unable to be completed on pathology tissue. 

A custom 60-gene panel, comparable to the panel patients receive in usual clinical care for genetic cancer risk assessment, will be used for genetic testing (Table 2). The commercial laboratory will perform genetic testing, variant confirmation, and variant interpretation. The report will include both pathogenic and likely pathogenic variants; variants of uncertain significance (VUS) will not be included. Variants will be reported to ClinVar [34] and sequence data will be deposited into dbGaP [35].

#### 2.5.2. Result Disclosure

Positive test results will be returned by phone with a genetic counselor. After the call, a copy of the test results and letter summarizing the follow-up recommendations will be provided to the participant or personal representative. Negative results will be returned by letter. All results, both positive and negative, will be placed in the participant’s medical record if they are living and a current member of the health plan. As appropriate, the study team will coordinate next steps in the participant’s care. If we are unable to reach a participant or personal representative to disclose results, we will place all results in the medical record.

#### 2.5.3. Cascade Testing

**Figure 2:**
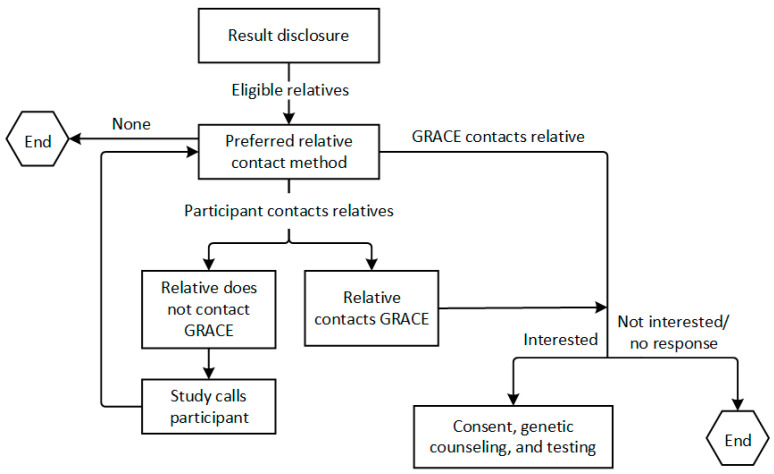
GRACE cascade testing flow.

The genetic counseling session will include collection of family history, and for any participants with positive results, cascade testing for the familial variant will be offered to first- and second-degree relatives at no cost within 90 days of result disclosure to the participant, which is offered by the commercial laboratory. Participants will be provided a letter to share with their family members. The current standard of care is that invitations to cascade testing are patient-mediated (i.e., the patient informs their own relatives). However, these approaches are known to be only partially effective in reaching all relatives; and evidence from outside the U.S. suggests that direct contact approaches may reach more relatives [36,37]. The GRACE study’s approach to cascade testing will be to encourage and support participant-mediated contact of relatives, but to be responsive to participant preferences and provide additional assistance with contacting relatives, including contacting relatives directly, as requested. We plan to allow for a variety of ways of approaching at-risk family members for cascade testing (Figure 2). We will let the participant or consenting family member drive the process in a way that best fits their preferences given differing family structures and communication styles, and we will not assume that a “one size fits all” approach will work.

Once we have spoken to an at-risk family member and know they are interested in having testing for the familial variant, the family member will complete the electronic consent form electronically in REDCap or by mail, depending on their preference. Once consent is complete, the study will schedule a pre-test genetic counseling session. At the completion of the genetic counseling session, the study will have a saliva test kit sent directly to the family member from the laboratory. Results disclosure will be carried out by the study genetic counselor when testing is complete if the results are positive, and if negative, a letter will be sent. We will follow the same approach for returning results as described above for participants. If the family member is a KPNW or KPCO member, we will place their results in their medical record.

#### 2.5.4. Data Collection

Genetic testing eligibility, uptake, and results as well as availability of pathology specimens for genetic testing will be captured for all patients. For at-risk family members of patients with positive findings, we will capture outreach and uptake. Using tumor registry searches and EHR chart review, we will also collect patient-specific data, including vital status, age at outreach, age at diagnosis, cancer stage at diagnosis, race/ethnicity, and insurance type. Study data will be collected and managed using REDCap electronic data capture tools hosted at KPNW [32,33].

#### 2.5.5. Interviews

Using a mixed purposeful sampling method [38], we will conduct semi-structured telephone interviews of approximately 20 at-risk family members who are eligible for cascade testing to assess perspectives on perceived risks, benefits, and preferred communication of being identified and offered genetic testing (Table 3). We will intend to interview an equal number of family members from both living and deceased participants and from each site. At-risk family members will be invited to participate in a one-hour interview near the time of consent or declining. Interviewees will be compensated USD 50 for their time.

### 2.6. Data Analysis

#### 2.6.1. Evaluate the Feasibility of Ovarian Cancer Traceback Testing

We will assess a set of feasibility metrics (Table 4) to quantify loss of patients across distinct points in the traceback testing to identify potential barriers and provide data points for feasibility assessment.

To test the hypothesis that key health system and patient-level factors associated with receipt of surgical care will be associated with availability of surgical pathology specimens, we will apply multivariable logistic regression, or other appropriate statistical modelling, to model the association between availability of pathology tissue for all eligible patients and predictors such as patient age at diagnosis, cancer stage, health system (KPNW vs. KPCO), race/ethnicity, time since diagnosis, and insurance type.

To test the hypothesis that contact rates and uptake of genetic testing of the patient’s pathology tissue will be lower for patients who are deceased, we will use multivariate logistic regression, or other appropriate statistical modelling, to model two dichotomous outcomes for each patient: (1) ability to contact the living patient or personal representative, and (2) uptake of genetic testing for the patient’s sample (among the subset of eligible patients where we are able to make contact with the patient or family members). The main predictor in these models will be participant vital status (living vs. deceased). Additional predictors will include variables such as time since diagnosis, cancer stage, insurance type, and participant demographics (e.g., age, race/ethnicity, socio-economic status). To test the hypothesis that uptake of cascade testing will be lower among at-risk family members of patients who are deceased, we will focus analyses on the subset of patients with a positive result. We will perform a chi-square analysis, or other appropriate statistical modelling such as multivariate analyses, to assess the association between the number of at-risk family members who received genetic testing and participant vital status. For the above analyses, a *p*-value of 0.05 will be used as the threshold to indicate statistical significance.

We estimated power for multivariate logistic regression models assuming 200 participants, a 2-sided test, and *p* < 0.05. For binary predictors, we will have 80% power to detect an odds ratio as low as 2.2. For continuous predictors, we will have 80% power to detect an odds ratio as low as 1.5. For chi-square tests to assess rates of cascade testing uptake, we will have 80% power to detect a small effect size (ω = 0.1) in uptake between participants who are deceased and participants who are living.

#### 2.6.2. Stakeholder Perspectives

Qualitative data analysis obtained from stakeholder interviews will be conducted using Dedoose (https://www.dedoose.com/ accessed on 1st October 2021). We will analyze the data using modified grounded theory and open coding in which inductively derived codes emerge from the data. This will be supplemented with axial coding through which we will identify codes deductively through literature review. Through memoing, coded data will be examined for themes that relate to ethical concepts such as privacy, autonomy, data sharing, clinical benefit, and others that may emerge through our analysis.

## 3. Discussion

Ovarian cancer traceback testing approaches have the potential to address a significant care gap in identifying individuals and families at increased genetic risk for cancer. Genetic counseling and testing rates in ovarian cancer patients are known to be low [39]. Given that approximately 80% of women are diagnosed with metastatic disease, the course of disease can be short and leave little time for genetic testing to occur [40]. The use of archived pathology specimens, typically stored by health care systems for clinical purposes, allows the opportunity to provide important genetic risk information to at-risk biological relatives even if the index patient is deceased. This allows for implementation of risk-reducing cost-effective interventions, such as increased surveillance and prophylactic surgery, to reduce cancer-related morbidity and mortality, including in biological relatives who have not yet developed cancer but are found to carry cancer risk variants [20,41].

The feasibility of implementing traceback programs to identify and recruit individuals with a prior diagnosis of ovarian cancer is currently unclear [42,43]. Feasibility could be impacted by multiple factors, such as patient interest and uptake, implementation approaches, and legal and regulatory guidelines at the federal, state, and health care system level [44,45,46]. Given contact may happen years after the cancer diagnosis, some patients may not have up-to-date contact information available, whereas others may not want to be identified and contacted for genetic testing [47]. Accuracy of tumor registry data at some health care systems may impact the ability to identify eligible patients. In cases where the patient is deceased, feasibility relies both on the availability of an archived pathology specimen for genetic testing and the ability to contact the personal representative to provide consent for genetic testing. Even in cases where the personal representative can be contacted, the personal representative may not be a biological relative who could benefit from the genetic risk information which may impact uptake, and they may not know the biological relatives to inform them. A traceback testing approach also raises legal and regulatory questions around who can provide consent for genetic testing when a patient is deceased and who can receive a patient’s genetic test results and facilitate cascade testing [46]. Though traceback testing programs offer great promise, potential challenges need to be identified and characterized to inform feasibility and optimize implementation approaches.

The GRACE study will provide critical information on the feasibility of implementing a traceback testing approach at the health care system level that leverages tumor registries and pathology specimens for genetic testing. This study will also provide important insights into how families weigh perceived benefits of a traceback testing approach compared with potential privacy concerns and the amount of information needed for decision-making. We will also collect information related to experience with the traceback process that could be applied to future approaches. Very little has been written on the legal and ethical implications of traceback testing to date. Through a critical review and analysis of the existing laws and literature pertaining to the processes and ethical issues involved in traceback testing, we will make a significant contribution to the emerging ethics literature on this subject. This work will undoubtedly serve as an important foundation for future work and approaches to traceback testing.

Although there are other approaches for successful traceback testing programs not based within health care systems, such as in foodborne illness outbreaks and infectious disease contact tracing [48,49,50], the benefit of the GRACE study’s approach to use stored pathology specimens is that it can provide important genetic risk information for biological relations of women who are now deceased. Given existing federal and state laws [51] that require retention of pathology specimens for a certain amount of time for clinical and oncological purposes, it is possible for other health care systems to implement similar programs. However, important learnings from the GRACE study will be to identify potential challenges and alternatives to this approach. Pathology specimens will not be available for all ovarian cancers, in particular those that are so advanced at diagnosis that surgery is not performed. The GRACE study will explore the availability of alternative biospecimens for deceased patients, such as additional pathology specimens from prior surgeries.

Overall, the GRACE study will inform broad implementation of such programs across health care systems, providing lifesaving information to prevent and mitigate the burden of ovarian cancer. In addition, these findings will inform the feasibility of traceback testing approaches for other populations at risk of hereditary cancer.

## Figures and Tables

**Table 1:** Genetic Risk Assessment in Ovarian Cancer (GRACE) inclusion and exclusion criteria.

Study Participants	
Inclusion Criteria	Exclusion Criteria
Female sex in EHR. One of the following ICD-O codes documented between 2008 and 2019: ○ C481 Peritoneum, specified parts; ○ C482 Peritoneum, NOS; ○ C569 Ovary; ○ C570 Fallopian tube. No evidence of prior genetic testing in the EHR or prior genetic testing for *BRCA1/2* only with a negative result or variants of uncertain significance (VUS). Must have available pathology tissue at a KPNW or KPCO affiliated hospital from resection/excision or have a biobanked sample if the patient is too sick to provide consent, in hospice care, or deceased or be living and able to submit a saliva sample. Age 18 years or older. Patient needs to be a KPNW or KPCO member at the time of ovarian cancer diagnosis. The patient does not need to be a current KPNW or KPCO member. Available personal representative to provide consent for testing of the patient’s pathology tissue or biobanked sample if the patient is too sick to provide consent or in hospice care or deceased.	Prior diagnosis of a hereditary cancer syndrome. Not a KPNW or KPCO member at the time of diagnosis. Unable to consent in English (KPCO only). Unable to provide informed consent. Opted out of research activities.
Cascade testing	
Inclusion criteria	Exclusion criteria
First or second degree relative of a study participant with a pathogenic or likely pathogenic variant. Age 18 years or older at the time they are approached for testing.	Known carrier of the same variant identified in the patient. Unable to consent in English (KPCO only).

**Table 2:** GRACE gene list.

*APC*	*FH*	*NF2*	*SDHB*
*ATM*	*FLCN*	*NTHL1*	*SDHC*
*AXIN2*	*GREM1*	*PALB2*	*SDHD*
*BAP1*	*HOXB13*	*PDGFRA*	*SMAD4*
*BARD1*	*KIT*	*PMS2*	*SMARCA4*
*BMPR1A*	*MAX*	*POLD1*	*SMARCB1*
*BRCA1*	*MEN1*	*POLE*	*STK11*
*BRCA2*	*MET*	*PRKAR1A*	*TEME127*
*BRIP1*	*MITF*	*PTCH1*	*TP53*
*CDC73*	*MLH1*	*PTEN*	*TSC1*
*CDH1*	*MSH2*	*RAD51C*	*TSC2*
*CDK4*	*MSH3*	*RAD51D*	*VHL*
*CDKN2A*	*MSH6*	*RB1*	
*CHEK2*	*MUTYH*	*RET*	
*DICER1*	*NBN*	*SDHA*	
*EPCAM*	*NF1*	*SDHAF2*	

**Table 3:** Interview domains.

Topics	Domains
Background knowledge and baseline opinions	Culture/kinship ties, practices, and beliefs about hereditary disease risk. Genetic literacy. Perceived risks and benefits of genetic testing. Decision-making process.
Participant’s experience in the study	Response to method of contact. Preferences for communication of familial risk. Decision-making process. Perceived risks and benefits of genetic testing. Preferences for communication of personal risk.

**Table 4:** Feasibility metrics.

Accuracy of tumor registries and pathology reports to identify patients with a correct diagnosis
Number of deceased patients with contact information for next of kin in EHR
Success rate to locate patients or contact next of kin for deceased patients
Uptake of genetic testing among contacted patients or next of kin
Availability of archived pathology specimens for germline genetic testing
Uptake of cascade testing among at-risk relatives
